# LH level on trigger day, number of follicles ≥16 mm but <18 mm, and number of retrieved oocytes are independent risk factors for polyspermy in gonadotropin-releasing hormone antagonist protocol

**DOI:** 10.3389/fendo.2024.1521734

**Published:** 2024-12-23

**Authors:** Qiongyu Wu, Lina He

**Affiliations:** Department of Reproductive Health and Infertility, Zigong Maternal and Child Health Hospital, Zigong, Sichuan, China

**Keywords:** polyspermy, IVF, GnRH antagonist, LH, follicle size, oocytes

## Abstract

**Introduction:**

The polyspermy rate is a quality control indicator in the embryology laboratory, and factors affecting polyspermy are of great interest. The gonadotropin-releasing hormone (GnRH) antagonist protocol is currently the mainstream protocol in most reproductive centers. This study explored the factors influencing polyspermy in *in vitro* fertilization (IVF) using the GnRH antagonist protocol and considered corresponding improvement measures.

**Methods:**

This retrospective case-control study analyzed 354 patients who underwent conventional IVF with a GnRH antagonist protocol at Zigong Maternal and Child Health Hospital from November 2019 to September 2023. Patients were divided into two groups based on the occurrence of polyspermy, and baseline characteristics and clinical data were compared between the groups. Variables with P<0.05 in univariate logistic regression were included in the multivariate logistic regression model. Cutoff values for variables with P<0.05 were calculated.

**Results:**

Multivariate logistic regression corrected for confounding factors identified that luteinizing hormone (LH) level on trigger day, the number of follicles ≥16 mm but <18 mm, and the number of retrieved oocytes were significantly associated with polyspermy (OR=1.305, P=0.005; OR=1.235, P=0.002; OR=1.101, P<0.001, respectively). The cutoff values were 1.95 IU/L, 4.5 follicles, and 16.5 oocytes, respectively.

**Conclusion:**

In the GnRH antagonist cycle, LH level on trigger day, the number of follicles ≥16 mm but <18 mm, and the number of retrieved oocytes are independent risk factors for polyspermy. When LH level on trigger day exceeds 1.95 IU/L, the number of follicles ≥16 mm but <18 mm exceeds 4, and the number of oocytes retrieved exceeds 16, the risk of polyspermy increases significantly.

## Introduction

Polyspermy refers to the penetration of an oocyte by two or more sperm during fertilization, resulting in multiple pronuclei. In natural conception, the physiological screening process within the female reproductive tract limits the number of sperm reaching the oocyte. This physiological decrease in sperm concentration is considered a mechanism to prevent polyspermy (1, 2). However, during conventional *in vitro* fertilization (IVF), sperm do not undergo the natural screening process of the uterus and fallopian tubes, resulting in a large number of active sperm being directly exposed to the oocyte. Additionally, controlled ovarian stimulation (COS) retrieves significantly more oocytes than a natural cycle does, and not all the oocytes are mature. Consequently, the likelihood of polyspermy is considerably increased (3). The occurrence of polyspermy can negatively affect laboratory parameters and pregnancy outcomes, such as reduced fertilization rates, lower high-quality embryo rates, decreased implantation rates, and lower clinical pregnancy rates, while increasing the risk of miscarriage (4–6). According to the 2017 Vienna Consensus (7), the polyspermy rate should be controlled within 6%.

Substantial research has focused on factors contributing to polyspermy in oocytes, such as the absence of zona pellucida 1 (ZP1) glycoprotein, defects in the transglutaminase 2 (Tgm2) gene, and abnormalities in cortical granule translocation (8–10). In contrast, there is less research on sperm-related factors. Li et al. found that mutations in the brain expressed X-linked protein 1 (BEX1) gene and a reduction in phospholipase C (PLC)-zeta are associated with an increased incidence of polyspermy (11). During IVF, factors such as oocyte quality and quantity, sex hormone levels in follicular fluid and serum, sperm concentration, polyploid sperm, and fertilization conditions all influence the incidence of polyspermy (12). Animal experiments have fully demonstrated that excessive ovarian stimulation negatively affects the quality of oocytes, reduces embryonic development potential and may increase the incidence of chromosomal abnormalities (13–15). Esther et al. also showed in their study that the dose of gonadotropin (Gn) was positively correlated with the aneuploidy rate (16). Therefore, an appropriate Gn dose and hormonal balance are essential to retrieve high-quality oocytes during COS.

While recent clinical studies on polyspermy in human IVF cycles remain limited, some research has investigated its contributing factors. For example, Sun et al. explored the factors associated with polyspermy in the ultra-long protocol (17). In their other study, as well as in various domestic and international studies, they analyzed the relationship between the number of oocytes retrieved and the incidence of polyspermy (18–21).

In IVF cycles, the gonadotropin-releasing hormone (GnRH) antagonist works by competitively blocking GnRH receptors in the anterior pituitary, thereby preventing the release of luteinizing hormone (LH) and follicle-stimulating hormone (FSH) triggered by endogenous GnRH (22). Compared to the GnRH agonist long protocol, the antagonist protocol is more cost-effective, time-efficient, and significantly reduces the risk of ovarian hyperstimulation syndrome (OHSS). Additionally, it yields oocytes of comparable quality and similar pregnancy outcomes, making it widely used in reproductive centers (23–26). However, research on polyspermy in IVF cycles using this protocol is still limited. This study aims to explore these factors to reduce polyspermy incidence and improve embryo transfer outcomes.

## Materials and methods

### Patients

This retrospective case-control study analyzed 354 patients who underwent conventional IVF with the GnRH antagonist protocol at Zigong Maternal and Child Health Hospital between November 2019 and September 2023. Patients were divided into two groups based on polyspermy occurrence: the normal fertilization group (n=198) and the polyspermy group (n=156).

Inclusion criteria (1): First IVF treatment at our hospital. (2) GnRH antagonist protocol. Exclusion criteria: (1) Rescue intracytoplasmic sperm injection (ICSI) treatment. (2) Male infertility or sperm donation. (3) Decreased ovarian reserve function, defined as antral follicle count (AFC) <5~7 or anti-Müllerian hormone (AMH) <0.5~1.1 μg/L. (4) Female age ≥40 years old. (5) Chromosomal abnormalities in either spouse. (6) Recurrent miscarriage patients, etc. This study was reviewed and approved by the Ethics Committee of Zigong Maternal and Child Health Hospital (202403).

### Ovulation stimulation and oocyte retrieval

Patients received gonadotropin starting on days 2-3 of menstruation until ovulation induction. Five to six days later, or when dominant follicle diameter >12 mm and serum estradiol (E_2_) >300 ng/L, 0.25 mg GnRH antagonist (Cetrotide, Merck Serono, Germany) was administered daily until the trigger day. Ovulation was induced with 250 μg of recombinant human chorionic gonadotropin (r-HCG, Ovidrel, Merck Serono, Germany) when the diameter of three follicles reached 17 mm, or two follicles reached 18 mm, or when the number of follicles with a diameter greater than 16 mm exceeded two-thirds. Oocytes were retrieved 36 hours post-trigger, and the cumulus-oocyte complex was cultured *in vitro* for 4 hours before fertilization. Corpus luteum support was initiated on the first day.

### Follicle measurement

To minimize measurement errors, all follicle measurements were conducted by the same physician. Each follicle’s largest cross-sectional area was recorded using two perpendicular diameters, and the arithmetic mean of these measurements was calculated for use.

### Semen treatment and short-term insemination

Abstinence for 2 to 5 days was required prior to oocyte retrieval. Semen samples were collected on the day of oocyte retrieval and processed using either density gradient centrifugation or the swim-up method. The separation medium for density gradient centrifugation consisted of Spermient 40% and 80% (Cook, Australia), while the washing and swim-up fluids were buffered fallopian tube fluid culture medium (ART-1023, Cooper Surgical, Inc.). Microdroplet insemination was performed, with each microdroplet prepared using 50 µL of G-IVF™ PLUS (Vitrolife, Sweden), covered with oil, and equilibrated overnight.

Forty hours after the r-HCG injection, the mixed sperm supernatant was added to the microdroplet at a concentration of 1×10^4^ motile sperm per oocyte. After confirming sperm motility and concentration under a microscope, the oocytes were added to the microdroplets, with 1 to 2 oocytes per droplet.

### Denuding and fertilization observation

Four to five hours after IVF fertilization, a 140 mm oocyte stripping needle is used to remove the granules. The oocytes are then transferred to cleavage medium (CM, Cook, Australia), which has been equilibrated overnight. The discharge of the second polar body is observed under a microscope. When the number of oocytes with two polar bodies reaches one-third or more of the mature oocytes, the oocytes are returned to the incubator for further culture. Fertilization is assessed after 18 hours. Oocytes with two pronuclei are considered normally fertilized, while those with three or more pronuclei are classified as polyspermy.

### Observation indicators

The baseline characteristics for the patients included female age, infertility duration, infertility type, infertility factors, proportion of polycystic ovary syndrome (PCOS), body mass index (BMI), basal serum levels of FSH, LH, E_2_, testosterone, progesterone, prolactin (PRL), and AMH. The clinical data indicators included Gn starting dose, Gn total dose, duration of Gn used, serum levels of E_2_, progesterone and LH on trigger day, the number of follicles with a diameter ≥ 14 mm but <16 mm, the number of follicles with a diameter ≥16 mm but <18 mm, the number of follicles larger than 18 mm, as well as the number of oocytes retrieved and Metaphase II (MII) oocytes.

### Statistical methods

SPSS 26.0 was used for statistical analysis. The measurement data were skewed and expressed as the median (25th percentile, 75th percentile) [M (P25, P75)]. The independent-samples Mann-Whitney U test was used for inter-group comparisons. Count data were expressed as composition ratios and percentages (%), and the chi-square test was applied for inter-group comparisons. If the sample size exceeded 40 and the expected frequency was above 5, Pearson’s chi-square test was applied. Conversely, if the sample size was under 40 or the expected frequency was below 5, Fisher’s exact test was preferred. Polyspermy occurrence was treated as the dependent variable, and indicators with P < 0.05 in the univariate logistic regression analysis were included in a multivariate logistic regression analysis (Forward-Wald method). The odds ratio (OR) and 95% confidence interval (CI) were calculated. Additionally, the cutoff value for variables with P < 0.05 in the multivariate logistic regression model was determined. A P-value < 0.05 was considered statistically significant.

## Results

### Baseline characteristics

A total of 354 antagonist cycles (354 patients) were included in this study, including 198 normal fertilization cycles (normal fertilization group) and 156 polyspermic fertilization cycles (polyspermic fertilization group). The total number of oocytes retrieved was 4682, and the polyspermy rate was 5.6% (266/4682). The age of the polyspermic fertilization group was significantly lower, while basal serum testosterone, AMH levels and proportion of PCOS were significantly higher. There was a significant difference in the distribution of infertility factors between the two groups. Other data showed no significant differences (P > 0.05), as shown in **Table 1**.

**Table 1:** Comparison of baseline characteristics and clinical data between the two groups.

Factors	Polyspermy group (n=156)	Normal fertilization group (n=198)	p value
Baseline characteristics			
Female age (year)	30.0 (28.0, 33.0)	31.0 (28.0, 34.0)	0.048
Infertility duration (year)	3.0 (2.0, 4.7)	3.0 (2.0, 5.0)	0.394
BMI (kg/m^2^)	21.4 (19.9, 23.6)	21.0 (19.5, 23.4)	0.247
Basal serum FSH (IU/L)	6.9 (5.8, 8.0)	7.3 (6.1, 8.5)	0.080
Basal serum E_2_ (ng/L)	42.8 (32.4, 50.4)	40.8 (31.8, 50.1)	0.364
Basal serum progesterone (ng/ml)	0.5 (0.4, 0.7)	0.5 (0.3, 0.7)	0.459
Basal serum LH (U/L)	4.1 (3.0, 6.4)	3.9 (2.8, 5.4)	0.050
Basal serum testosterone (ng/dl)	0.3 (0.2, 0.4)	0.3 (0.2, 0.3)	0.020
Basal serum PRL (ng/ml)	18.5 (15.1, 24.5)	18.0 (12.7, 23.0)	0.102
AMH (ng/ml)	4.6 (2.7, 7.3)	3.2 (2.0, 4.6)	<0.001
Infertility type			0.201
Primary infertility (%)	80/156 (51.3)	88/198 (44.4)	
Secondary infertility (%)	76/156 (48.7)	110/198 (55.6)	
Infertility factors			0.017
Pelvic and fallopian tube (%)	125/156 (80.1)	158/198 (79.8)	
Ovulatory dysfunction (%)	21/156 (13.5)	12/198 (6.1)	
Endometriosis (%)	3/156 (1.9)	6/198 (3.0)	
Others (%)	7/156 (4.5)	22/198 (11.1)	
PCOS (%)	45/156 (28.8)	14/198 (7.1)	<0.001
Clinical data			
Gn starting dose (U)	225.0 (187.5, 225.0)	225.0 (225.0, 300.0)	0.014
Gn total dose (U)	1912.5 (1575.0, 2250.0)	2006.3 (1621.9, 2400.0)	0.164
Duration of Gn used (days)	9.0 (8.3, 10.0)	9.0 (8.0, 10.0)	0.631
E_2_ level on trigger day (ng/L)	3515.0 (2565.0, 4960.0)	2350.0 (1625.0, 3525.0)	<0.001
LH level on trigger day (U/L)	2.0 (1.3, 3.0)	1.8 (1.3, 2.5)	0.126
Progesterone level on trigger day (ng/ml)	1.1 (0.8, 1.4)	0.8 (0.6, 1.2)	<0.001
Number of follicles >14 mm, <16mm (n)	3.0 (2.0, 6.0)	3.0 (1.0, 4.0)	0.020
Number of follicles >16mm, <18mm (n)	4.0 (2.0, 6.0)	3.0 (1.0, 4.0)	<0.001
Number of follicles >18mm (n)	3.0 (2.0, 4.0)	3.0 (2.0, 4.0)	0.378
Number of oocytes retrieved (n)	16.0 (10.0, 21.0)	10.0 (6.0, 13.8)	<0.001
MII oocytes (n)	13.0 (8.0, 18.0)	8.0 (5.0, 11.0)	<0.001

BMI, body mass index; FSH, follicle-stimulating hormone; E_2_, estradiol; LH, luteinizing hormone; PRL, prolactin; AMH, anti-Müllerian hormone; PCOS, polycystic ovary syndrome; Gn, gonadotropin; MII, Metaphase II.

The polyspermy group consists of 156 females (n = 156), while the normal fertilization group comprises 198 females (n = 198). Values are presented as the median (25th percentile, 75th percentile) [M (P25, P75)] or percentages (%). The independent-samples Mann-Whitney U test and chi-square test were applied as appropriate.

### Clinical data

The Gn starting dose in the polyspermic fertilization group was significantly lower, while the E_2_ and progesterone levels on trigger day, the number of follicles with diameter ≥14 mm but <16mm, the number of follicles with diameter ≥16 mm but <18mm, the number of retrieved oocytes, and MII oocytes were significantly higher. No significant differences were observed in the other data (P > 0.05), as shown in **Table 1**.

### Logistic regression analysis of factors influencing polyspermy

A multivariate logistic stepwise regression model was established, with variables including female age, basal serum LH and testosterone, AMH, infertility factors, proportion of PCOS, Gn starting dose, Gn total dose, E_2_ and LH levels on trigger day, the number of follicles ≥14 mm but <16 mm, the number of follicles ≥16 mm but <18 mm, retrieved oocytes, and MII oocytes. These variables were selected based on P < 0.05 in the univariate logistic regression analysis.

**Figure 1:**
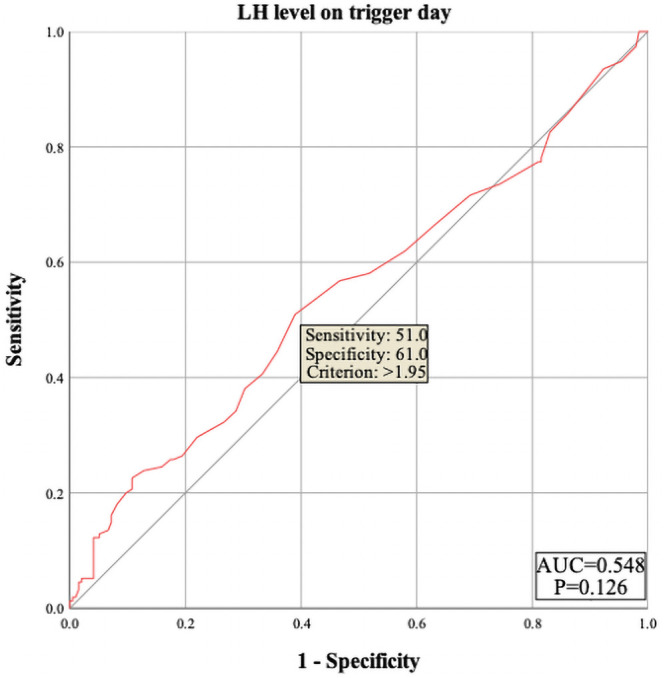
ROC curve of luteinizing hormone (LH) level on trigger day to predict polyspermy. The area under the curve (AUC) for LH level on trigger day to predict polyspermy is 0.548, with a cutoff value of 1.95 U/L. The sensitivity is 51.0%, and the specificity is 61.0%.

**Figure 2:**
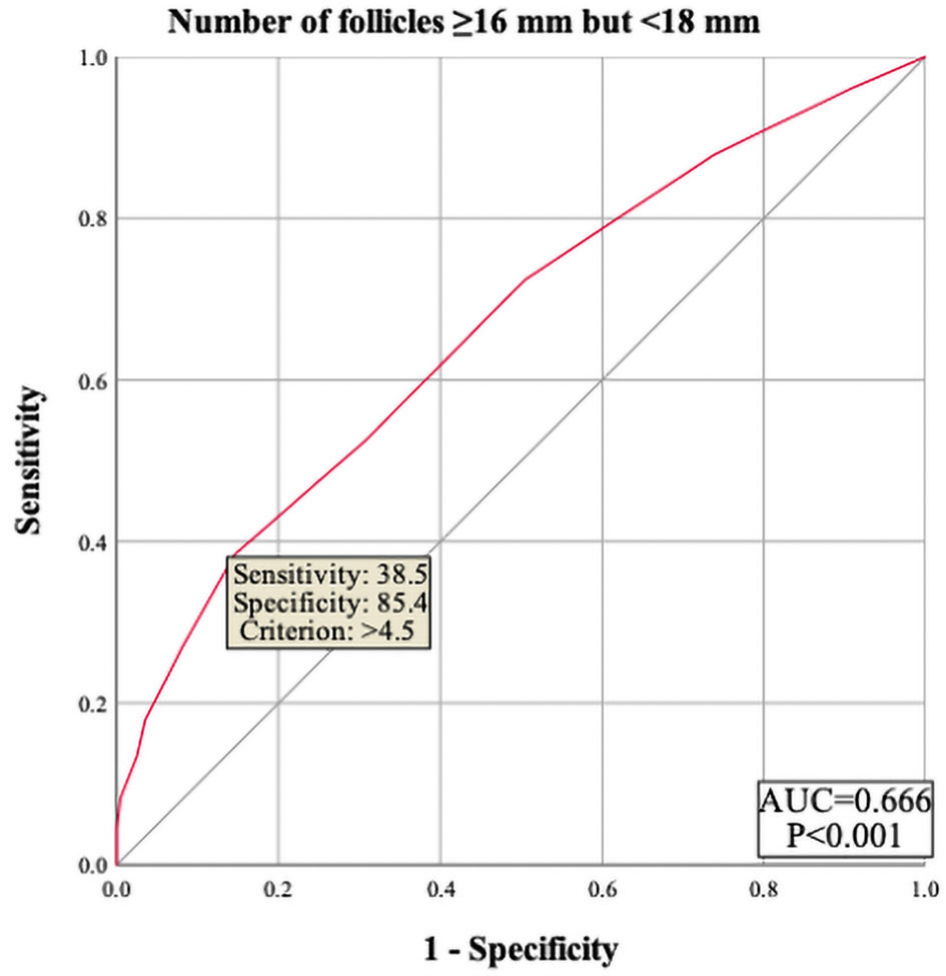
ROC curve of number of follicles ≥16 mm but <18 mm to predict polyspermy. The area under the curve (AUC) for number of follicles 216 mm but <18 mm on trigger day to predict polyspermy is 0.666, with a cutoff value of 4.5 follicles. The sensitivity is 38.5%, and the specificity is 85.4%.

**Figure 3:**
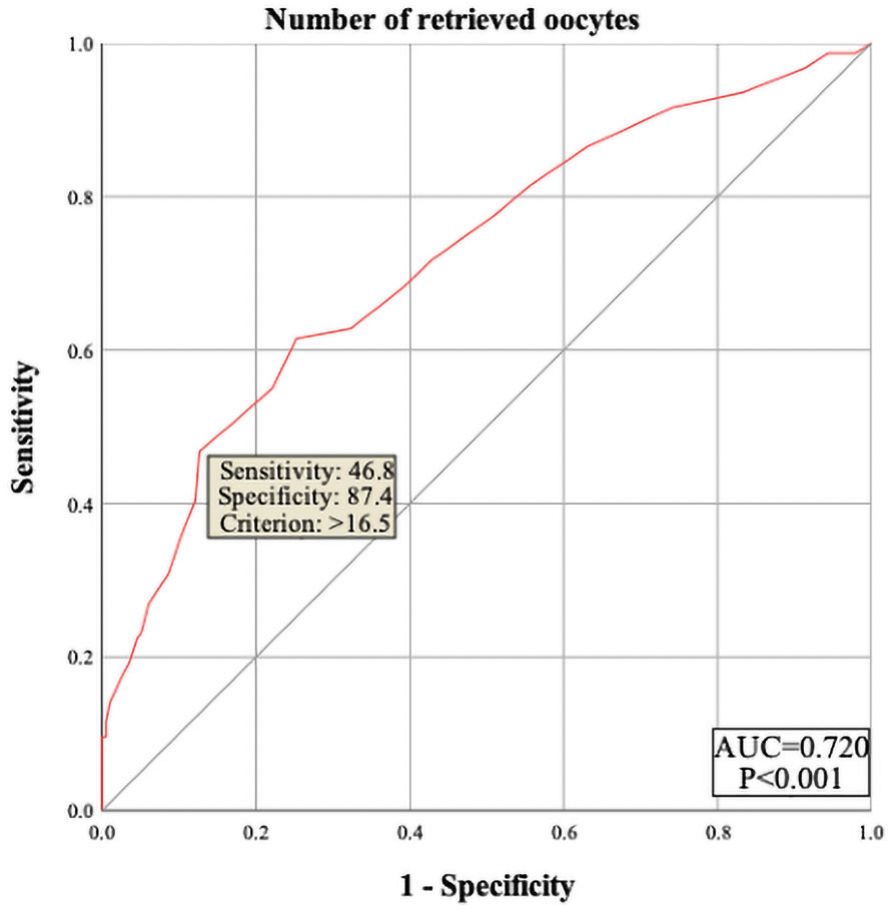
ROC curve of number of retrieved oocytes to predict polyspermy. The area under the curve (AUC) for number of retrieved oocytes on trigger day to predict polyspermy is 0.720, with a cutoff value of 16.5 oocytes. The sensitivity is 46.8%, and specificity is 87.4%.

The results showed that female age, basal serum LH and testosterone, AMH, infertility factors, PCOS, Gn starting dose, Gn total dose, E_2_ level on trigger day, the number of follicles ≥14 mm but <16 mm, and MII oocytes did not enter the regression equation (P > 0.05), indicating that they are not independent factors influencing polyspermy. However, LH level on trigger day, the number of follicles ≥16 mm but <18 mm, and the number of retrieved oocytes were significantly associated with the occurrence of polyspermy (OR=1.305, P=0.005; OR=1.235, P=0.002; OR=1.101, P<0.001, respectively), as shown in **Table 2**. Their cutoff values were 1.95 IU/L, 4.5 follicles, and 16.5 oocytes, as shown in **Figures 1**–**3** respectively.

**Table 2:** Logistic regression analysis of factors related to polyspermy rate.

Factors	Univariate regression			Multiple regression		
	P value	OR	95%CI	P value	OR	95%CI
Female age (year)	0.050	0.946	0.896-1.000			
Infertility duration (year)	0.829	1.009	0.929-1.096			
BMI (kg/m^2^)	0.686	1.015	0.943-1.094			
Basal serum FSH (IU/L)	0.079	0.910	0.819-1.011			
Basal serum E_2_ (ng/L)	0.260	1.004	0.997-1.010			
Basal serum progesterone (ng/ml)	0.134	1.194	0.947-1.505			
Basal serum LH (U/L)	0.015*	1.107	1.020-1.201			
Basal serum testosterone (ng/dl)	0.024*	5.420	1.247-23.567			
Basal serum PRL (ng/ml)	0.156	1.017	0.994-1.041			
AMH (ng/ml)	0.000*	1.207	1.115-1.307			
Infertility Type						
Primary infertility (%)	0.194	1.323	0.868-2.017			
Secondary infertility (%)		1.000				
Infertility factors	0.022*					
Pelvic and fallopian tube (%)	0.043*	2.486	1.029-6.008			
Ovulatory dysfunction (%)	0.003*	5.500	1.817-16.646			
Endometriosis (%)	0.586	1.571	0.309-7.989			
Others (%)		1.000				
PCOS (%)	0.000*	0.188	0.099-0.358			
Gn starting dose (U)	0.013*	0.995	0.991-0.999			
Gn total dose (U)	0.028*	1.000	0.999-1.000			
Duration of Gn used (days)	0.756	0.977	0.842-1.133			
E_2_ level on trigger day (ng/L)	0.000*	1.000	1.000-1.000			
LH level on trigger day (U/L)	0.039*	1.172	1.008-1.364	0.005	1.305	1.082-1.574
Progesterone level on trigger day (ng/ml)	0.053	1.430	0.996-2.054			
Number of follicles >14 mm, <16mm (n)	0.000*	1.147	1.069-1.230			
Number of follicles >16mm, <18mm (n)	0.000*	1.346	1.211-1.497	0.002	1.235	1.081-1.412
Number of follicles >18mm (n)	0.308	1.055	0.952-1.169			
Number of oocytes retrieved (n)	0.000*	1.132	1.092-1.174	0.000	1.101	1.054-1.150
MII oocytes(n)	0.000*	1.136	1.092-1.182			

OR, odds radio; CI, confidence interval; BMI, body mass index; FSH, follicle-stimulating hormone; E_2_, estradiol; LH, luteinizing hormone; PRL, prolactin; AMH, anti-Müllerian hormone; PCOS, polycystic ovary syndrome; Gn, gonadotropin; MII, Metaphase II.

* indicates p < 0.05 in the univariate logistic regression analysis.

## Discussion

Oocyte quality is a critical factor affecting polyspermy. Mature oocytes are capable of preventing the entry of two or more sperm. When a sperm penetrates a mature oocyte, cortical granules, which contain proteases, are released into the perivitelline space. This causes the oocyte membrane or ZP to harden, preventing other sperm from binding to the ZP and thus inhibiting polyspermy (27–29). Cortical granules in human oocytes are closely associated with polyspermy. Abnormal release of cortical granules in oocytes leads to an inability to prevent polyspermy. As the cytoplasm of the oocyte ages, its ability to be activated and continue development decreases, which may impair the normal cortical reaction. Additionally, immature oocytes fail to release cortical granules after sperm penetration, leading to incomplete oocyte activation. As a result, immature oocytes are more prone to polyspermy compared to mature ones (3, 30).

GnRH agonists bind to their receptors and induce pituitary suppression, effectively down-regulating hormone levels prior to COS. This allows for better follicular uniformity in patients. In the GnRH antagonist cycle, however, the physiological rise in FSH level during the luteal-follicular transition triggers the development of heterogeneous follicles, resulting in a slightly lower maturation rate compared to the agonist cycle (31). Additionally, antagonists competitively occupy GnRH receptors in a short period of time during COS. The resulting unrestrained FSH may lead to asynchronous development of FSH-sensitive follicles, potentially reducing the number of mature oocytes obtained (32, 33).

We conducted a multi-factor logistic regression analysis to control for confounding factors, and the results indicated that only LH level on trigger day, the number of follicles measuring ≥16mm but <18mm and the number of oocytes retrieved were all independent risk factors for polyspermy. Among these, the number of oocytes retrieved had the greatest impact on the occurrence of polyspermy. Our findings are consistent with several domestic and international studies, which also show a positive correlation between the number of oocytes and the rate of polyspermy (18, 19, 21). However, Sun et al., in their study on the antagonist protocol, found that when the number of oocytes is 15 or fewer, an increasing number of oocytes is associated with a higher risk of polyspermy. Yet, when the number of oocytes exceeds 15, the risk of polyspermy does not further increase (20). This differs from our conclusion, which we speculate may be due to the inclusion of patients with varying ovarian functions in their study.

In our research, the number of follicles larger than 18 mm and the number of MII oocytes had no significant relationship with polyspermy. However, the total number of oocytes retrieved and the number of follicles ≥16 mm but <18 mm were found to be independent risk factors for polyspermy. Specifically, when these numbers exceed 16 and 4 respectively, the risk of polyspermy increases. This may be because follicles ≥18 mm are more likely to produce oocytes with synchronized nuclear and cytoplasmic maturation, whereas follicles measuring 16–18 mm may produce a higher proportion of oocytes with mature nuclei but immature cytoplasm. Kahraman et al. also speculated in their study that small follicles (those <17 mm in diameter) containing MII oocytes may have nuclear competence but not necessarily cytoplasmic competence (31). This suggests that during COS, it is advisable to wait until more follicles grow to 18 mm before retrieval to reduce the likelihood of abnormal fertilization. Many studies have demonstrated that smaller follicles yield fewer mature oocytes, although a certain proportion of mature oocytes can still be retrieved from small follicles (34, 35).

In clinical practice, physicians often puncture follicles smaller than 18 mm during oocyte retrieval, partly to prevent OHSS and partly to increase the number of oocytes to enhance the patient’s chances of pregnancy. If immature oocytes are observed under the microscope after retrieval, *in vitro* maturation (IVM) can be performed prior to fertilization to reduce the risk of polyspermy.

The correlation between various indicators during COS and polyspermy has been a focus for researchers. Tavaniotou et al. (36) found that patients with elevated LH levels had fewer MII oocytes and lower pregnancy rates. Additionally, pregnant patients showed lower LH levels on the trigger day compared to non-pregnant patients. LH regulates signaling pathways and has a significant impact on oocyte quality (37, 38). In another study, Sun et al. (17) identified LH level below 1 U/L on trigger day in the ultra-long protocol as an independent risk factor for polyspermy. Based on this finding, they recommended moderate LH supplementation during COS in the ultra-long protocol to reduce the risk of polyspermy—an approach that contrasts with our findings in the antagonist protocol. This discrepancy may be attributed to the deep pituitary suppression in the ultra-long protocol.

LH plays a critical role in follicular development, and moderate LH supplementation could potentially improve oocyte quality (39). Westergaard et al. (40) conducted a study on 200 women undergoing ovarian stimulation with the standard long protocol and found that 48% of the women still had severely suppressed LH levels (<0.5 IU/l) on the 8th day of COS. In contrast, in the antagonist protocol, approximately 80% of LH surges occur before the antagonist is administered. Furthermore, while GnRH antagonist starts acting as quickly as 4 hours after injection, their suppression of the pituitary is not as potent as that of GnRH agonists, meaning LH may not be fully suppressed.

In our study, we observed a positive correlation between LH level on trigger day and the incidence of polyspermy, suggesting that elevated LH level may affect oocyte quality in the antagonist protocol. However, there is currently no global consensus on optimal LH control. Our analysis showed that in the antagonist protocol, when LH level on trigger day exceed 1.95 IU/L, the rate of polyspermy increases. These findings suggest that greater attention should be given to pre-treatment before COS, along with flexible administration of antagonists during COS, to better manage LH level on trigger day and reduce their potential impact on oocyte quality.

As this study is based on real-world data, there are inherent baseline imbalances among the patients. Furthermore, being a retrospective analysis, the study has certain limitations. While we employed multivariate logistic regression to adjust for confounding factors, some potential confounders may have been overlooked. Therefore, well-designed, multicenter, prospective randomized controlled trials (RCTs) are needed to further validate our findings.

## Data Availability

The raw data supporting the conclusions of this article will be made available by the authors, without undue reservation.

## References

[1] Sakkas D Ramalingam M Garrido N Barratt CL. Sperm selection in natural conception: what can we learn from Mother Nature to improve assisted reproduction outcomes? Hum Reprod Update. (2015) 21:711–26. doi: 10.1093/humupd/dmv042 PMC4594619 26386468

[2] Leung ETY Lee CL Tian X Lam KKW Li RHW Ng EHY. Simulating nature in sperm selection for assisted reproduction. Nat Rev Urol. (2022) 19:16–36. doi: 10.1038/s41585-021-00530-9 34741158

[3] Wang WH Day BN Wu GM. How does polyspermy happen in mammalian oocytes? Microsc Res Tech. (2003) 61:335–41. doi: 10.1002/jemt.10346 12811738

[4] Chen W Bai H Li M Xue X Shi J. Effects of three pro-nuclei (3PN) incidence on laboratory and clinical outcomes after early rescue intracytoplasmic sperm injection (rescue-ICSI): an analysis of a 5-year period. Gynecol Endocrinol. (2021) 37:137–40. doi: 10.1080/09513590.2020.1757640 32342711

[5] Li M Zhang S Shi W Ren W Liu Y Tang Q. Effects of three pro-nuclei (3PN) proportion incidence on clinical outcomes of patients with lower retrieved oocytes in the fresh cleavage-stage embryo transfer (ET) cycles. Gynecol Endocrinol. (2016) 32:891–5. doi: 10.1080/09513590.2016.1190330 27251984

[6] Figueira RC Setti AS Braga DP Iaconelli A Jr. Borges E Jr. Prognostic value of triploid zygotes on intracytoplasmic sperm injection outcomes. J Assist Reprod Genet. (2011) 28:879–83. doi: 10.1007/s10815-011-9610-0 PMC3220437 21805146

[7] ESHRE Special Interest Group of Embryology and Alpha Scientists in Reproductive Medicine. The Vienna consensus: report of an expert meeting on the development of ART laboratory performance indicators. Reprod BioMed Online. (2017) 35:494–510. doi: 10.1016/j.rbmo.2017.06.015 28784335

[8] Cui Z Lu Y Miao Y Dai X Zhang Y Xiong B. Transglutaminase 2 crosslinks zona pellucida glycoprotein 3 to prevent polyspermy. Cell Death Differentiation. (2022) 29:1466–73. doi: 10.1038/s41418-022-00933-0 PMC9345939 35017645

[9] Banks N Avella M Baibakov B Tokuhiro K Dean J. ZP1 contributes to the prevention of polyspermy in mice. Fertility Sterility. (2016) 106:e347. doi: 10.1016/j.fertnstert.2016.07.985

[10] Cheeseman LP Boulanger J Bond LM Schuh M. Two pathways regulate cortical granule translocation to prevent polyspermy in mouse oocytes. Nat Commun. (2016) 7:13726. doi: 10.1038/ncomms13726 27991490 PMC5187413

[11] Li X Hou J Shan X Tian E Wang Y Xu W. P–257 An unknown cause lead to polyspermy in IVF cycles and 0PN zygotes in ICSI cycles in male patient. Hum Reprod. (2021) 36. doi: 10.1093/humrep/deab130.256

[12] Suzuki H Saito Y Kagawa N Yang X. *In vitro* fertilization and polyspermy in the pig: factors affecting fertilization rates and cytoskeletal reorganization of the oocyte. Microsc Res Tech. (2003) 61:327–34. doi: 10.1002/jemt.10345 12811737

[13] Chang MC. Digynic triploidy after superovulation. Nature. (1977) 266:382–3. doi: 10.1038/266382b0 859609

[14] Ertzeid G Storeng R. The impact of ovarian stimulation on implantation and fetal development in mice. Hum Reprod. (2001) 16:221–5. doi: 10.1093/humrep/16.2.221 11157810

[15] Van der Auwera I D'Hooghe T. Superovulation of female mice delays embryonic and fetal development. Hum Reprod. (2001) 16:1237–43. doi: 10.1093/humrep/16.6.1237 11387298

[16] Baart EB Martini E Eijkemans MJ Van Opstal D Beckers NG Verhoeff A. Milder ovarian stimulation for *in-vitro* fertilization reduces aneuploidy in the human preimplantation embryo: a randomized controlled trial. Hum Reprod. (2007) 22:980–8. doi: 10.1093/humrep/del484 17204525

[17] Sun YT Wang J Li XX Zhu AZ. Influencing factors of polyspermy during conventional *in vitro* fertilization in super-long protocol. Chin J Of Reprod And Contraception. (2023) 43:169–75. doi: 10.3760/cma.j.cn101441-20211108-00494

[18] Li M Zhao W Xue X Zhang S Shi W Shi J. Three pro-nuclei (3PN) incidence factors and clinical outcomes: a retrospective study from the fresh embryo transfer of *in vitro* fertilization with donor sperm (IVF-D). Int J Clin Exp Med. (2015) 8:13997–4003. PMC4613043 26550358

[19] Zhao J Li Y Liu D Liu NH Chen XH Yao ZY. Relationship between polypronuclear and E2, P on the day of HCG injection,Oocyte number, insemination sperm concentration, fertilization rate, pregnancy rate. Life Sci Res. (2010) 14:54–6. doi: 10.16605/j.cnki.1007-7847.2010.01.015

[20] Sun Y Zhu A. Correlation between the number of oocytes and the increase of polyspermy rate in IVF cycles. Gynecol Endocrinol. (2023) 39:2217270. doi: 10.1080/09513590.2023.2217270 37247634

[21] Passaro C Rossiter C Tsagdi S Armstrong E Barrie A. Campbell A. P-263 Clinical factors influencing the incidence of tripronuclear zygotes in IVF. Hum Reprod. (2022) 37. doi: 10.1093/humrep/deac107.252

[22] Gillies PS Faulds D Balfour JA Perry CM. Ganirelix. Drugs. (2000) 59:107–11; discussion 12-3. doi: 10.2165/00003495-200059010-00007 10718102

[23] Jing M Lin C Zhu W Tu X Chen Q Wang X. Cost-effectiveness analysis of GnRH-agonist long-protocol and GnRH-antagonist protocol for *in vitro* fertilization. Sci Rep. (2020) 10:8732. doi: 10.1038/s41598-020-65558-0 32457475 PMC7251086

[24] Toftager M Bogstad J Løssl K Prætorius L Zedeler A Bryndorf T. Cumulative live birth rates after one ART cycle including all subsequent frozen-thaw cycles in 1050 women: secondary outcome of an RCT comparing GnRH-antagonist and GnRH-agonist protocols. Hum Reprod. (2017) 32:556–67. doi: 10.1093/humrep/dew358 28130435

[25] Cota AM Oliveira JB Petersen CG Mauri AL Massaro FC Silva LF. GnRH agonist versus GnRH antagonist in assisted reproduction cycles: oocyte morphology. Reprod Biol Endocrinol. (2012) 10:33. doi: 10.1186/1477-7827-10-33 22540993 PMC3464873

[26] Orvieto R. Stop GnRH-agonist/GnRH-antagonist protocol: a different insight on ovarian stimulation for IVF. Reprod Biol Endocrinol. (2023) 21:13. doi: 10.1186/s12958-023-01069-7 36710334 PMC9885692

[27] Gardner AJ Evans JP. Mammalian membrane block to polyspermy: new insights into how mammalian eggs prevent fertilisation by multiple sperm. Reprod Fertil Dev. (2006) 18:53–61. doi: 10.1071/rd05122 16478602

[28] Nishio S Emori C Wiseman B Fahrenkamp D Dioguardi E Zamora-Caballero S. ZP2 cleavage blocks polyspermy by modulating the architecture of the egg coat. Cell. (2024) 187:1440–59.e24. doi: 10.1016/j.cell.2024.02.013 38490181 PMC10976854

[29] Bianchi E Wright GJ. Sperm meets egg: the genetics of mammalian fertilization. Annu Rev Genet. (2016) 50:93–111. doi: 10.1146/annurev-genet-121415-121834 27617973

[30] van der Ven HH Al-Hasani S Diedrich K Hamerich U Lehmann F Krebs D. Polyspermy in *in vitro* fertilization of human oocytes: frequency and possible causes. Ann N Y Acad Sci. (1985) 442:88–95. doi: 10.1111/j.1749-6632.1985.tb37508.x 3860066

[31] Kahraman S Cetinkaya CP Cetinkaya M Yelke H Colakoglu YK Aygun M. The effect of follicle size and homogeneity of follicular development on the morphokinetics of human embryos. J Assist Reprod Genet. (2017) 34:895–903. doi: 10.1007/s10815-017-0935-1 28470453 PMC5476546

[32] Fanchin R Méndez Lozano DH Schonäuer LM Cunha-Filho JS Frydman R. Hormonal manipulations in the luteal phase to coordinate subsequent antral follicle growth during ovarian stimulation. Reprod BioMed Online. (2005) 10:721–8. doi: 10.1016/s1472-6483(10)61115-7 15970000

[33] Kolibianakis EM Albano C Kahn J Camus M Tournaye H Van Steirteghem AC. Exposure to high levels of luteinizing hormone and estradiol in the early follicular phase of gonadotropin-releasing hormone antagonist cycles is associated with a reduced chance of pregnancy. Fertil Steril. (2003) 79:873–80. doi: 10.1016/s0015-0282(02)04920-8 12749423

[34] Shapiro BS Rasouli MA Verma K Raman A Garner FC Aguirre M. The effect of ovarian follicle size on oocyte and embryology outcomes. Fertil Steril. (2022) 117:1170–6. doi: 10.1016/j.fertnstert.2022.02.017 35367061

[35] McCulloh DH Kutchukhidze N Charkviani T Zhorzholadze T Barbakadze T Munné S. Follicle size indicates oocyte maturity and blastocyst formation but not blastocyst euploidy following controlled ovarian hyperstimulation of oocyte donors. Hum Reprod. (2020) 35:545–56. doi: 10.1093/humrep/dez291 32142586

[36] Tavaniotou A Albano C Van Steirteghem A Devroey P. The impact of LH serum concentration on the clinical outcome of IVF cycles in patients receiving two regimens of clomiphene citrate/gonadotrophin/0.25 mg cetrorelix. Reprod BioMed Online. (2003) 6:421–6. doi: 10.1016/s1472-6483(10)62161-x 12831586

[37] Arroyo A Kim B Yeh J. Luteinizing hormone action in human oocyte maturation and quality: signaling pathways, regulation, and clinical impact. Reprod Sci. (2020) 27:1223–52. doi: 10.1007/s43032-019-00137-x PMC7190682 32046451

[38] Zamah AM Hsieh M Chen J Vigne JL Rosen MP Cedars MI. Human oocyte maturation is dependent on LH-stimulated accumulation of the epidermal growth factor-like growth factor, amphiregulin. Hum Reprod. (2010) 25:2569–78. doi: 10.1093/humrep/deq212 PMC2939758 20719813

[39] Bosch E Alviggi C Lispi M Conforti A Hanyaloglu AC Chuderland D. Reduced FSH and LH action: implications for medically assisted reproduction. Hum Reprod. (2021) 36:1469–80. doi: 10.1093/humrep/deab065 PMC8129594 33792685

[40] Lambalk CB Banga FR Huirne JA Toftager M Pinborg A Homburg R. GnRH antagonist versus long agonist protocols in IVF: a systematic review and meta-analysis accounting for patient type. Hum Reprod Update. (2017) 23:560–79. doi: 10.1093/humupd/dmx017 28903472

